# Differentiation of Remedies

**Published:** 1885-08

**Authors:** P. P. Wells

**Affiliations:** Brooklyn, N. Y.


### HOMŒOPATHIC PHYSICIAN,

A MONTHLY JOURNAL OF MEDICAL SCIENCE.

"If our school ever gives up the strict inductive method of Hahnemann, we are lost, and deserve only to be mentioned as a caricature in the history of medicine."—CONSTANTINE HERING.

Vol. V. AUGUST, 1885. No. 8.

### DIFFERENTIATION OF REMEDIES.

P. P. WELLS, M. D., BROOKLYN, N. Y.

(*Continued from page 232.*)

The actions of these three drugs on the male genital organs and functions are neither numerous nor very important as therapeutic indices. In this relation they may help to a right selection when the due consideration of the sick phenomena of other organs and their functions have, by reason of their obscurity or similarity, left the prescriber in doubt as to the right selection. It will be seen on the record Acon. has itching on the prepuce, which only disappears momentarily when rubbed; Bell. has itching and tickling in the glans; Bry. has stitching and burning itching on the prepuce and red, itching rash on the glans.

Acon. has stitch and pinching in the glans when urinating; Bell. has stitches in the retracted testicles; Bry., stitch in the testicles while sitting.

Acon. has pains in the testicles as if crushed; Bell. has tearing in the spermatic cord, in the evening, in bed; Acon. has crawling in the genital organs (it has also amorous paroxysms and diminished or increased sexual instinct, alternating with torpidity); Bell. has loss of sexual instinct and perfect indifference to all incitements of it. Pollutions without erections; and it has them twice in a night. Escape of prostatic fluid, with relaxed penis.

On the female organs and functions, we find Acon. has copious catamenia in plethoric subjects; suppressed catamenia in lively, full-blooded young persons who live a sedentary life; madness on the appearance of the catamenia; hæmorrhage from the uterus.

Bell. has too copious catamenia, and delayed for four, eight, or twenty days, or anticipating four days; is too pale; suppressed; hæmorrhage from the uterus at other times than the catamenial period; discharge of offensive smelling, bright-red blood in clots. [Sabin. similar, but fluid.]

Bry. has suppressed catamenia, with bleeding from the nose; catamenia is anticipating; returns in eight, fourteen, or twenty-one days; pinching in the distended abdomen, as if the catamenia would appear; hæmorrhage from the uterus (not at the catamenial period); during the catamenia, tearing pains in the limbs; the blood is dark, with pains in back and head.

Acon. has copious, thick, yellowish leucorrhœa; Bell. has leucorrhœa, with pain in the abdomen; Bry. has little relation to this affection.

Before the catamenia, Bell. has fatigue, pain in the abdomen, loss of appetite, and obscured vision. During the catamenia, nocturnal sweat on the chest, with yawnings and cold shuddering on the back; anxiety of the heart; great thirst; squeezing, tearing—now in the back and now in the arms. Neither of the others are charged with symptoms at this time.

Bell. has strong pressing to the genitals, as if all would be forced out [see Lil., Tig., and Sep.], with subsequent discharge of blood or mucus; internal shootings; great dryness of the vagina. Bry. has swelling of the labia majora, with black, hard pustule therein.

The uterine hæmorrhages of these three drugs are characterized by the blood of Acon. being dark colored; Bry. has it both dark and bright, while that which is most characteristic of Bell. is a mixture—the one color being clotted, the other fluid. Thus it will be seen Acon. has bleeding from the veins, Bell. and Bry. from both veins and arteries, that of Bell. being mixed and partly clotted, while that of Bry. is either the one or the other.

Acon. is credited with important symptoms during the pregnant state—such as fear of death; shooting, burning, excoriating pains in the liver; nausea and vomiting, with pain in the stomach, and, after each meal, with pain in the head. The other two are not charged with analogous symptoms.

Acon. acts on the mammary gland to increase its secretion; in this it has Bry. for a companion; Bell. has erysipelatous inflammation of the gland and Bry. rheumatic.

Acon. has catarrhal attacks and coryza, also with headache, pain in the abdomen, ringing in the ears, and copious urine. (Given in the initiation of such catarrhal attacks, Acon. will often cut them short. It is worth observing and remembering in how many acute affections where Acon. is curative its usefulness is almost wholly limited to the first stage of such sickness, while in the latter it is nearly wholly powerless for good. This is well illustrated in cases of croup or other laryngeal or tracheal affections with which we are now engaged.) Morning hoarseness, cracked voice; attacks like paralysis of the epiglottis, with choking, ringing in the trachea (*eingesihofentreitsgeful*) as if asleep, said of hands or feet.

Bell. has catarrh with oppression of the chest; coryza, cough, especially at night, with stitches in the sternum; voice rough and hoarse, weak and piping, nasal; loss of voice; great painfulness of the larynx, with danger of suffocation while feeling it and turning the neck, coughing, speaking, and when inspiring; spasmodic constriction of the larynx; rattling of mucus in the bronchi, inflammation of the larynx and trachea.

Bry. has hoarseness and rough voice while walking in the open air; with cough and rattling in the chest; with inclination to sweating. In the morning oppression of the chest, as if loaded with mucus. Tenacious mucus in the throat, which is removed by slight cough. Hawking up of yellow mucus from the throat.

Acon. has cough in the hot stage of fever; short, dry cough, with constant inclination to it from tickling in the larynx, especially excited by smoking tobacco or by drinking, or it returns after midnight, every half hour; spasmodic, rough, cracked cough, also with danger of suffocation and constriction of the larynx; cough, with thick, white or bloody mucous expectoration; dry cough, with general heat, thirst and great restlessness; cough after every cold taken, which is especially troublesome at night; dry cough, which permits no rest evenings, with constant irritation and oppression of the upper half of the left lung; hæmoptysis, without pain, but with nocturnal anxiety, constant complaining and whimpering, fearfulness, red face, better while lying down; painful sensitiveness of the larynx to touch.

Bell. Incitement to cough by irritation of the larynx from every inspiration; in the epigastrium, as if there were something lying there. Cough, with flow of thick saliva at midday, with subsequent heat; with tightness and congestion of the chest; with taste of blood in the mouth; with shooting pain in the side. Cough at night, from evening to ten o'clock, in quarterhour attacks of three or four coughs; severe in sleep with grinding of the teeth, which wakes from sleep. Dry cough in the forenoon, very severe, as from a foreign body in the larynx, with coryza; short cough, evenings, in bed, from tickling or itching in the larynx; with oppressed chest, as if from dry catarrh. Day and night from tickling in the throat pit, or with headache and redness of the face. Slight cough from scratching in the throat. Scraping, hollow cough; spasmodic, especially after midnight, with retching. Before the cough, crying (whooping cough) or pain in the stomach with the cough, pressure in nape of the neck, as if it would break, trembling in the stomach as if he would vomit. After cough, sneezing.

Bry. has short, dry, hacking cough, as if from mucus in the trachea, especially while smoking tobacco, or with pressure and excruciating pain in the larynx, after the cough, with inclination to deep inspiration, in the evening in bed, with scratching as if from roughness and dryness in the larynx. Dry cough, as if from the stomach, with crawling and tickling in the epigastrium, especially in the morning, with flow of water from the mouth like water brash. Spasmodic cough, from smoking tobacco, after eating and drinking, with vomiting of what has been swallowed; with children, especially after midnight, with spasmodic, suffocating oppression of the breathing before the cough. Cough with expectoration, excited by crawling in the throat; in the forenoon, yellow expectoration; in the morning, in bed, with copious mucous expectoration of dirty reddish mucus; expectoration of blood, of clots or of clear blood, or of bloody streaked mucus. With the cough, stitches in the head, the epigastrium, the hypochondria, the sternum, chest or sides, in the throat; sensation as if the head or chest would burst. Pain in the epigastrium as if raw; sneezing, heaving as if he would vomit; pressure which passes through the head.

Acon. has expectoration of dark blood, streaked with blood; purulent, yellow, slimy, whitish, sticky, putrid tasting and sweetish.

Bell. has bloody, mixed, bright and dark, and fluid in clots, purulent, yellow, slimy, insipid tasting, salt, sour, disgusting, and offensive smelling expectoration.

Bry. has insipid, putrid, sour, disgusting.

*Respiration.*—Acon. has short, especially in sleep, after midnight or on rising from lying down; interrupted, through the nose, especially in sleep; offensive breath, anxious, difficult, sighing, or rapid and superficial, or loud, strong, and noisy, with open mouth, with tightness of the chest, slow breathing in sleep; suffocating attacks, with anxiety, tightness, squeezing (*Beklemmung*), and sense of contraction in the chest, and anxiety in the chest which impedes respiration, with warm sweat on the forehead.

Bell. has difficult breathing; small, anxious, short, and rapid, also with groaning, irregular, now short, now slow, now natural, and then as if he had drawn his last breath (this irregularity is quite characteristic), violent expiration, short breath after drinking.

Bry. has hard, rapid, anxious breathing, or it is wholly hindered by stitches in the chest, which compel sitting up; short breath from pressure and sensation of warmth in the epigastrium, with rapid expiration; with anxiety, as if from the abdomen in the morning; slow, deep respiration, especially during exertion; need of deep breath from a sensation of want of breath and obstruction in the chest, with pain during deep inspiration as if something forced itself out; laborious breathing, with exertion of the abdominal muscles, and single, deep inspirations; sighing respirations.

*Pains in the chest.*—Acon. has pressing pain in the chest, which is only relieved for a short time by bending the chest backward; contractive pain in the chest, as if the ribs were drawn against each other; sense of heaviness in the chest, as if it were pressed together from all sides; stitching pressure on the right side of the sternum; pinching, digging in the right side; shootings and stitch in the chest and sides of the chest, especially when breathing and coughing; often with whimpering, complaining disposition, or with anxiety or fretfulness, or with oppressed breathing; bruised pain in the lower ribs, or in the middle of the sternum, is much increased by pressure, of which the patient complains much; painful crawling in the chest, as if from insects; pressing together of the chest in the region of the heart.

Bell. has squeezing of the chest, with pressure in the epigastrium, which rises to the throat and obstructs the respiration and makes it anxious, and with retching; spasmodic squeezing (*Beklemmung*) in the epigastrium while walking, and compelling deep inspiration; severe, as if the chest would be crushed in; in the evening in bed, with difficult breathing, as if from mucus in the chest, with burning pressure in the chest; between the shoulders, with short breath; in the right half, also with anxiety; in the sternum over the ensiform cartilage; outward at the sixth rib; squeezing pressure in both sides of the chest; shootings and stitches in the sternum when coughing and yawning; under the clavicle when walking; in the left side, worse when moving; in the right side, also especially toward evening, with oppressed breathing; cutting, as if from knives, near the ensiform cartilage; stitching, pinching in the upper part of the sternum, and on both sides of it; pressing stitch in the left rib cartilages, with burning during expiration; pressing, cutting in the right side; tightness of the chest; heat in the chest, extending from the abdomen; burning in the right half of the chest.

Bry. has attacks of tightness of the chest, especially at night, with stitches in the abdomen and urging to stool; oppression of the chest, with stitches in the evening (better after discharge of wind), with sensation as of rising up, which embarrasses breath and speech, with pressure in the epigastrium, with stitches in the side; pressure on the chest, on the sternum, especially when walking in the open air or when breathing. especially with ice-cold feet, as if from mucus in the chest, with stitches in the sternum, in inspiration, less after eating, oppressive behind the sternum, worse while breathing; pressing stitches from within outward; heaviness in the chest, with heaviness of the body, better after eating; shooting and stitches in the chest when coughing and breathing, with deep inspiration especially, in the side, by starts, better in the open air, as if from a sore under the sternum; in the morning with each breath and when raising the arms, when turning in bed on the side not lain on, when lying on the back, and by every movement it is much increased, with throbbing in the lower part of the right chest; tensive pain in the chest while walking, in the great curve of the ribs toward the back, during inspiration, with stitch from deep inspiration and bending forward, especially under the shoulder-blades; sensation as if all were loose in the chest and would fall into the abdomen; oppressive pain in the chest, immediately over the epigastrium, while sitting bent forward and lying on the side (in bed), clawing together near the sternum; heat in the chest and in the face at the same time.

These three remedies have important actions on the central organ of circulation. Acon. has slow thrusts in the region of the heart; palpitation, with great anxiety; universal heat, especially in the face, and great debility of the limbs; palpitation of the heart in young, full-blooded, sensitive persons who lead a sedentary life.

Bell. has throbbing in the chest with great restlessness, under the sternum and above the epigastrium; palpitation, violent, with shaking in the neck and head, worse when moving, with difficult, slow breathing, vexing while ascending stairs; trembling of the heart, also with anxiety and pressing pain.

Bry. has very violent beating of the heart with squeezing tightness of the chest.

It will be noticed, if we carefully examine the symptoms of respiration of these drugs, that those of Acon. are largely characterized by defective innervation of the respiratory organs, by reason of which embarrassment of their especial function is the result from which often comes blood stosis in lung or pleural tissues, and this, if not relieved, often passes into acute inflammations, of which this stosis has represented the first stage. Experience has proved abundantly that it is just here, and only here, that Acon. has power to conquer these, and that in the early, congestive stage it is second in importance to no other remedy. It will also be found that it closely imitates that form of asthma which is made up of impeded or suspended passage of nerve-power to these organs, which presents a seeming paralytic condition of the motive power of these organs.

In a general view of the relations of Bell. to these organs and their functions, similar remarks might seem to be applicable by reason of the great similarity of the general aspect of the phenomena these drugs have produced by their action on these organs and their functions. But if examined specifically, the differences of the two will become sufficiently apparent. Compare the anxious, difficult sighing, or rapid and superficial, or the loud, strong, with open mouth, of Acon., with the small, anxious, short, and rapid, with groans, of Bell.; or its characteristic irregular respiration, now rapid, now slow, now normal, and now as drawing the last breath, and the differences are so many as to suggest a query as to the similarity. And yet these two drugs are closely and vitally related to affections of these organs by reason of their similar actions on them, as has been found in their provings on the healthy. And it is not a matter of indifference in a given case to be treated which of these drugs shall be selected. They do not belong to cases of lung or pleural inflammations or neurosis in a general manner, but each and only to such cases as are marked by the specific characteristics of that drug. Though a thousand successive cases may be met which for reason of the poverty of our language relating to them we must call by the same name, they do not therefore all call for the same curative. Each successive case calls only for that drug which has shown greatest similarity to its own sick phenomena. In treating sicknesses homœopathically, names cease to be things. Treating names (diagnosis) is the exclusive property of old physic. The homœopathist who finds himself so engaged should know he is trespassing on his neighbor's preserves, where he has no business, and of being found there he ought to be heartily ashamed. We have placed the groups of respiratory symptoms, as well as other groups, in close juxtaposition, that they might be the more conveniently compared and the differences controlling choice more readily appreciated.

It will be noticed as a peculiarity of the modifications of respiration by the action of Bry. that they are mostly accompanied by its characteristic pain—that of stitch, short breath from pressure, and heat in the epigastrium. The stitch or stab characteristic of Bry. is, as compared with that of Acon., as if it were made by a broader blade, that of Acon. being often so narrow as to become a fine pricking (in incipient pneumonia), while that of Squill is as if from a broad blade. As compared with that of Bell. it is a simple stitch, while that of this last is complex, as, for instance, it is a pinching or a pressing stitch, or it may be so expanded as to be more of a cutting character. Acon. is accompanied by its peculiar morale. This pain with both Bell. and Bry. is increased by motion.

*Back.*—Acon. has pains in the loins, also while walking, like labor pains, pressing pains in the left sacro-lumbar region (Kreutz); burning, corrosive pain on the right side near the vertebræ; severe shooting and digging, left side, downward, through the whole extent of the back to the sacro-lumbar region, increased greatly by inspiration; bruised and painful paralytic stiffness in the sacro-lumbar region and loins (often into the back and neck), often with tensive pressure and pain in the abdomen, as from flatulence; painful boring, left side near the sacro-lumbar region; crawling in the spine, as if from chaffers; cutting from the spine to the abdomen; digging, boring from the right shoulder-blade forward to the chest, increased by inspiration; weak sensation in the neck, as if the flesh were loose, and shootings on moving the head; stitches in the sides of the neck; externally, fine stitches in the neck; pressure in the left side of cervical vertebræ, or on the neck inward to the trachea; painful stiffness of the neck.

Bell. has a squeezing pain in the sacro-lumbar region and coccyx, with difficult fast walking and lying, especially on the back, particularly at night, diminished by standing and slow walking; painful stiffness in the sacro-lumbar region after sitting, with inability to rise up; cutting outward on the margin of the sacrum when rising up from sitting; drawing in the circumference of the pelvis; pain in the ischia when sitting, as if there were no flesh on the bones; shooting in the spine as if from knives; pain like a sprain; gnawing also with cough; squeezing pressure with tension when standing erect; large, red, stitching pimples on the shoulder-blades and excruciating pain in the skin; abscess on the shoulder; pressure under the left shoulder-blade; drawing pressure and squeezing pinching between the right shoulder-blade and the vertebræ; pain as if sprained between the shoulder-blades; drawing of the shoulder-blades to the spine downward evenings; shooting and itching stitches on the shoulder-blades; painful stiffness of the neck as far as between the shoulder-blades, especially when turning the head and in the morning; pressure near the occiput; stitch while bending the head backward; pressure on the throat when feeling it or bending the head backward; painful swelling and stiffness, also of the nape; squeezing tension and drawing pressure in the muscles; sensible throbbing in the vessels; distended veins; sour sweat on the throat; swelling of the glands; painful swelling of the axillary gland; tearing in the axilla.

Bry. has pain in the small of the back, which makes walking very difficult; stitches also in the back, especially at night; bruised and cramp pains when sitting, especially when lying down, relieved by moving; painful stiffness, which will not permit walking erect; tearing in the back, especially in the lumbar vertebræ, particularly when standing, relieved when sitting, disappears when lying down, and which permits neither stooping nor bending; burning; stitches in the lumbar vertebræ; shooting, jerking in both sides of the spine, especially mornings and evenings when sitting; drawing downward in the back when sitting, relieved by moving; contractive, spasmodic pain across the back; spasmodic pain between the shoulder-blades, like a shuddering; burning; pressure, and at the same time on the chest when sitting, which disappears when walking. In the nape of the neck, pain, as if he had taken cold; painful weakness; painful stiffness and tension, and also in the throat when moving the head; drawing in the throat, which extends to the ear; excruciating pain in the neck, face, and muscles of mastication while moving the parts; sweat in the axilla; creeping from the axilla to the hip as if from a mouse.

On comparing the pains of these three remedies in the lower part of the back, Acon. is found to be like labor pains; neither of the others is so characterized. Its pains are more in the left side of the spine, but it has also burning, corrosive pains in the right side of the spine; neither of the others has. It has also, with some of its pains, its characteristic crawling sensation. Acon. has paralytic stiffness of these parts; Bell. has painful stiffness; that of Acon. often passes into the back and neck; that of Bell. is after sitting, making it difficult to rise up; Bry. prevents walking erect; Bell. has in this region squeezing pain with cough, worse from fast walking and lying on the back and at night—relieved by standing and slow walking (characteristic); it has also cutting and drawing pains in the margins of the pelvis bones.

Bry. has pains which make it difficult to walk; shooting pains, especially at night; it has pains like a bruise, and cramp when sitting, and especially when lying down; relieved by moving. In this and in one or two other symptoms of the spinal regions, Bry. is found departing from its general characteristic of relief by repose. It will be found that a few of its symptoms are aggravated by rest; its pains permit neither stooping nor bending; it has stitches and jerkings in the lumbar vertebræ, and especially on both sides of the spine, especially morning and evening, when sitting; it has tearing pains in the lumbar region; neither of the others has; it has shooting and jerkings on each side of the spine mornings and evenings, when sitting; Acon. has severe shooting and digging in the left side downward through the whole extent of the back to the lumbar region, increased greatly by inspiration; Bell. has shooting in the spine, as if from knives; Acon. has painful boring in the left side near the sacro-lumbar region, which neither of the others has; it only has crawling in the spine, and cuttings from the spine to the abdomen.

Bell. has pains like a sprain in the back, and gnawing, with cough, and squeezing pressure with tension when standing erect.

Bry. has drawing downward in the back when sitting; relieved by motion; spasmodic pain across the back.

In the shoulder-blades, Acon. has digging, boring pain from the right to the chest, increased by inspiration.

Bell. has pressure under the left shoulder-blade; drawing pressure and squeezing, pinching pain.

[TO BE CONTINUED.]

---

In the New York *Medical Record* for July 11th, a remarkable case is described of hiccough that resisted all methods of treatment until finally a small abscess formed in the epigastric region, from which the end of a needle protruded. This was discovered and extracted by the patient's sister. The physician, Dr. Liégeois, then made an incision and found eight more needles. The operation was followed by complete cure.

